# A higher circulating concentration of 25-hydroxyvitamin-D decreases the risk of renal cell carcinoma: a case-control study

**DOI:** 10.1590/S1677-5538.IBJU.2018.0186

**Published:** 2019-07-27

**Authors:** Fei Li, HongFan Zhao, Lina Hou, Fengsheng Ling, Yue Zhang, WanLong Tan

**Affiliations:** 1 Department of Urology, Nanfang Hospital, Southern Medical University, Guangzhou, Guangdong, P.R. China; 2 Department of Healthy Management, Nanfang Hospital, Southern Medical University, Guangzhou, Guangdong, P. R. China; 3 Department of Urology, Foshan Women and Children Hospital Foshan Guangdong, P.R. China

**Keywords:** Carcinoma, Renal Cell, Vitamin D, 25-Hydroxyvitamin D 2

## Abstract

**Objective:**

To investigate the relationship between vitamin D status, using circulating 25-hydroxyvitamin D [25 (OH) D], and renal cell carcinoma (RCC) risk in a case-control study, because the association between the two is unclear in China.

**Materials and Methods:**

A total of 135 incident RCC cases were matched with 135 controls by age and sex. The blood samples were collected on the first day of hospitalization before surgery to measure plasma 25 (OH) D. Logistic regression analyses were used to calculate odds ratios (ORs) and 95% confidence intervals (95% CIs) with adjustment for several confounders (e.g. age, gender, smoking and season of blood draw). Furthermore, the association of RCC with 25 (OH) D in units of 10 ng / mL as a continuous variable were also examined.

**Results:**

The average plasma 25 (OH) D concentrations in RCC were significantly lower compared with those of the controls (21.5 ± 7.4 ng / mL vs. 24.1 ± 6.6 ng / mL, respectively; P = 0.003). In the adjusted model, inverse associations were observed between circulating 25 (OH) D levels and RCC risk for 25 (OH) D insufficiency (20-30 ng / mL) with OR of 0.50 (95% CI: 0.29-0.88; P = 0.015) and a normal 25 (OH) D level (≥ 30 ng / mL) with OR of 0.30 (95% CI: 0.13-0.72; P = 0.007), compared with 25 (OH) D deficiency (< 20 ng / mL). Furthermore, results with 25 (OH) D as a linear variable indicated that each 10 ng / mL increment of plasma 25 (OH) D corresponded to a 12% decrease in RCC risk.

**Conclusions:**

This case-control study on a Chinese Han population supports the protective effect of a higher circulating concentration of 25 (OH) against RCC, whether the confounding factors are adjusted or not.

## INTRODUCTION

Renal cell carcinoma (RCC) is one of the most common adult malignancies, and the incidence of this disease has increased at a rate of approximately 1.6% per year over the last 10 years (1). In China, there were an estimated 66.800 new cases of RCC and 23.400 deaths in 2015 (2). Clear cell RCC, accounting for 70-80% of all cases, is the most common subtype of RCC. Approximately 50% of patients still suffer recurrence in spite of complete surgical resection. There are no established preventive measures against it, although certain studies have shown that smoking, hypertension, obesity, family history of the disease and genetic susceptibility may increase the risk of RCC (3). Therefore, feasible preventive measures for RCC are of considerable clinical and public health importance.

Vitamin D, the “sunshine” vitamin, is transformed by exposure to sunlight and obtained from certain foods, such as oily fish and cereals. The primary circulating form of vitamin D is 25-hydroxyvitamin D [(25 (OH) D], the accepted indicator of an individual's vitamin D status (4). It is well known that vitamin D initially plays important roles in promoting bone health through the regulation of bone growth and remodeling, as well as calcium homeostasis (4, 5). Vitamin D has been shown to protect against many types of cancer in the past few years. Some laboratory studies demonstrated that vitamin D could inhibit cancer cell proliferation, invasion, angiogenesis and metastasis (6, 7). To date, few studies have examined the association between vitamin D and kidney cancer. Furthermore, our previous meta-analysis indicated that higher levels of circulating 25 (OH) D could reduce the risk of kidney cancer by 21% (8). To our knowledge, the study of the relationship between circulating 25 (OH) D levels and RCC risk has been limited in Chinese populations.

In this study, the epidemiological association between plasma 25 (OH) D concentrations and the risk of RCC was examined within a retrospective case-control study. For this investigation, pre-diagnostic circulating concentrations of 25 (OH) D were measured and data were analyzed from 135 RCC cases and 135 controls.

## MATERIALS AND METHODS

In this hospital-based case-control study, all subjects were recruited from the Chinese Han population at Nanfang Hospital (Guangzhou, China) from January 2015 to January 2017. All subjects were informed about the purpose of the study and gave their written consent. Plasma samples from 135 RCC patients, matched with 135 controls by 10-year age group and sex, were sent to the Department of Clinical Laboratory of Nanfang Hospital on the first day of hospitalization before surgery to measure the levels of plasma 25 (OH) D. To avoid the influence of other factors, the levels of serum creatinine and calcium (Ca) were also measured. The Ethics Committee of Nanfang Hospital approved this study.

The inclusion criterion for the cases was histopathologically confirmed disease after surgery, no more than two months after the initial diagnosis of RCC. The diagnosis was conducted by two pathologists at Nanfang Hospital of Southern Medical University, according to the criteria of the World Health Organization classification of tumors (9).

The inclusion criteria for the controls were that they were apparently healthy, with no history of any type of cancer. Those with special dietary habits, those with an intake of vitamin D and calcium supplements within the previous three months, those with conditions that affected their sun exposure habits, those who had resided in other countries during the year prior to recruitment, and those who were pregnant or lactating, were not included in the study (10).

The patients and controls were interviewed to collect data on the following variables: age, sex, weight, height, smoking status, alcohol consumption status and use of vitamin D supplements in the past year. No participants had resided in other countries during the previous year, therefore the impact of sun exposure was not included in the final analysis. Furthermore, to account for seasonal variation affecting vitamin D status, season-specific were categorized based on the month of blood collection (“Winter months” = November-April and “Summer months” = May-October).

### Statistical analysis

Quantitative data [the plasma levels of 25 (OH) D] are described as the mean and standard deviation, and qualitative data are expressed as absolute and relative frequencies. Student's t-tests were used for the comparison of quantitative variables and the chi-square test was applied for qualitative variables. We used logistic regression analyses to calculate odds ratios (ORs) and 95% confidence intervals (95% CIs) for RCC in association with plasma levels of 25 (OH) D. Two models were used to examine the relationships. First, total 25 (OH) D concentrations (ng / mL) were divided into tertiles as follows: 25 (OH) D deficiency (< 20 ng / mL), insufficiency (20-30 ng / mL), and normal 25 (OH) D levels (≥ 30 ng / mL) (11). In a second model, the plasma 25 (OH) D concentration was included in units of 10 ng / mL as a continuous variable with a single linear term. In additional to plasma 25(OH) D variables, the common RCC risk factors were adjusted in our multivariate model analysis, including age, sex, body mass index (BMI), smoking status, alcohol consumption status, season of blood draw, hypertension and diabetes. Furthermore, we examined the associations between circulating levels of 25 (OH) D and the clinical features of RCC, such as tumor stage and metastasis. In this analysis, the ‘low stage’ group included cases of T1 and T2, while the ‘high stage’ group involved T3 and T4 cases, although the number of T3 and T4 cases was limited. All the collected data were statistically analyzed using STATA Statistical Software, version 15.0 (StataCorp, College Station, TX, USA). P < 0.05 was regarded as statistically significant.

## RESULTS

The characteristics of the cases and controls are presented in Table-1. There were 135 newly diagnosed patients with RCC and 135 controls included in our analysis. Of those 135 RCC cases, 92 cases were clear cell RCC and the remaining 43 cases were non-clear cell RCC. According to the TNM classification, the numbers of cases at T1 to T4 were 97, 23, 11 and 4, respectively. Furthermore, there were 18 RCC cases with metastasis. The average age was 53.28 years (SD =13.93) for the cases and 53.68 years (SD = 15.84) for the controls, with no significant difference observed. Furthermore, there were no significant differences found in sex, hypertension, diabetes, smoking status and alcohol consumption between the two groups, although differences were observed in height, weight and BMI over 30 kg / m^2^. BMI was higher among the cases compared with the controls (P = 0.045) (Table-1).

**Table 1:** Baseline characteristics of cases and controls.

Characteristics		Cases (N = 135)			Controls (N = 135)			P-value
		No	%	Mean ± SD	No	%	Mean ± SD	
**Sex**								
	Male	90	66.7		91	67.4		0.897
	Female	45	33.3		44	32.6		
Age				53.28 ± 13.93			53.68 ± 15.84	0.826
**Season of blood drawn**								
	Winter (Nov-Apr)	67	49.6	21.3 ± 7.4	78	57.8	23.0 ± 6.5	0.179
	Summer (May-Oct)	68	50.4	21.8 ± 7.4	57	42.2	25.7 ± 6.6	
Height (cm)				166.41 ± 7.71			162.86 ± 6.89	< 0.001
Weight (kg)				66.23 ± 11.30			61.05 ±1 0.09	< 0.001
Body mass index (kg / m^2^)				23.84 ± 3.53			23.00 ± 3.38	0.045
< 25.0		91	67.4	21.87 ± 2.11	97	71.9	21.41 ± 2.26	
25.0-29.9		36	26.7	27.21 ± 1.51	36	26.7	26.78 ± 1.41	
> 30.0		8	5.9	31.09 ± 1.35	2	1.5	32.25 ± 0.92	
**Hypertension**								
	Yes	45	33.3		37	27.4		0.290
	No	90	66.7		98	72.6		
**Diabetes**								
	Yes	14	10.4		17	12.6		0.567
	No	121	89.6		118	87.4		
**History of smoking**								
	Yes	37	27.4		35	25.9		0.783
	No	98	72.6		100	74.1		
**History of alcohol intake**								
	Yes	33	24.4		27	20		0.380
	No	102	75.6		108	80		
Ca (nmol / L)				2.23 ± 0.35			2.23 ± 0.22	0.961
Cr (nmol / L)				82.48 ± 33.82			78.34 ± 15.54	0.198
25-(OH) (ng / mL)				21.5 ± 7.4			24.1 ± 6.6	0.003

In our study, vitamin D deficiency was defined as 25 (OH) D < 20 mg / dL, and the sufficient range as > 30 mg / dL 45.9% of the cases and 30.4% of the controls had 25 (OH) D levels < 20 ng / mL 32.6% of the men and 49.4% of the women had 25 (OH) D levels < 20 ng / mL. Slightly higher 25 (OH) D concentrations were found in blood drawn from the patients in the summer compared with the winter (21.8 ± 7.4 vs. 21.3 ± 7.4, respectively), while the higher magnitude was enhanced in the controls for blood drawn in summer compared with the winter (25.7 ± 6.6 vs. 23.0 ± 6.5, respectively). There was no significant difference between the seasons of blood draw. Furthermore, there were significant differences in the circulating concentrations of 25 (OH) D in the different tumor stages (T1: 21.8 ± 7.3 ng / mL, T2: 23.9 ± 7.5 ng / mL, T3: 15.4 ± 5.1 ng / mL and T4: 19.3 ± 7.0 ng / mL; P = 0.014) (Table-2). Notably, no significant difference was detected in the cases with metastasis (20.4 ± 6.5 ng / mL) in comparison with the cases without metastasis (21.7 ± 7.5 ng / mL). Overall, the average plasma 25 (OH) D concentrations in RCC cases were significantly lower than those in the controls (21.5 ± 7.4 ng / mL compared with 24.1 ± 6.6 ng / mL, respectively; P = 0.003) (Table-1).

**Table 2:** The Circulating 25-(OH) D levels of renal cell carcinoma cases according to clinical features.

Clinical feature		Cases	25-(OH) D (ng / mL)	P-value
Pathological type		135		0.314
Clear cell carcinoma		92	21.1 ± 6.4	
Non-Clear cell carcinoma		43	22.5 ± 9.2	
Chromophobe		20	23.2 ± 10.2	
Papillary		10	25.1 ± 8.1	
Others		13	19.4 ± 7.9	
**T-stage**				**0.014**
	T1	97	21.8 ± 7.3	
	T2	23	23.9 ± 7.5	
	T3	11	15.4 ± 5.1	
	T4	4	19.3 ± 7.0	
**Metastasis**				
	Yes	18	20.4 ± 6.5	0.485
	No	117	21.7 ± 7.5	

In the entire study population, the crude ORs and 95% CIs compared with 25 (OH) D deficiency (< 20 ng / mL) were 0.57 (95% CI: 0.340.96; P = 0.034) for 25 (OH) D insufficiency (20-30 ng / mL), and 0.33 (95% CI: 0.15-0.75; P = 0.009) for a normal 25 (OH) D level (≥ 30 ng / mL). After further adjustment for age, sex, BMI, season of blood draw, diabetes, hypertension, smoking and alcohol consumption, this relationship was largely unchanged for 25 (OH) D insufficiency 0.50 (95% CI: 0.29-0.88; P = 0.015) and a normal 25 (OH) D level 0.30 (95% CI: 0.13-0.72; P = 0.007). The overall results indicated that an inverse association was observed between circulating 25 (OH) D levels and the risk of RCC. Furthermore, results with 25 (OH) D as a continuous, linear variable were similar, with both crude ORs and adjusted ORs of 0.88 (95% CI: 0.81-0.96; P = 0.003) and 0.87 (95% CI: 0.80-0.95; P = 0.002) respectively. The findings from the linear analysis showed that each 10 ng / mL increment of plasma 25 (OH) D concentration corresponded to a 12% decrease in RCC risk (Table-3).

**Table 3:** Association between 25 (OH) D and the risk of renal cell carcinoma in total, and according to tumor stage and metastasis.

Subjects		Circulating 25 (OH) D (ng / mL)			
		< 20	20-29.9	≥ 30	Per 10
All subjects	Controls	41	72	22	
	Cases	62	62	11	
	OR^a^ (95% CI)	Ref	0.57 (0.34-0.96)	0.33 (0.15-0.75)	0.88 (0.81-0.96)
	P-value^a^	Ref	0.034	0.009	0.003
	OR^b^ (95% CI)	Ref	0.50 (0.29-0.88)	0.30 (0.13-0.72)	0.87 (0.80-0.95)
	P-value^b^	Ref	0.015	0.007	0.002
T-stage	Low stage (T1 + T2)	50	59	11	
	High stage (T3 + T4)	12	3	0	
	OR^a^ (95% CI)	Ref	0.21 (0.06-0.79)	/	0.90 (0.84-0.97)
	P-value^a^	Ref	0.021	0.999	0.005
	OR^b^ (95% CI)	Ref	0.18 (0.04-0.79)	/	0.90 (0.84-0.97)
	P-value^b^	Ref	0.024	0.999	0.004
Metastasis	Yes / No	9 / 53	8 / 54	1 / 10	
	OR^a^ (95% CI)	Ref	0.87 (0.31-2.43)	0.59 (0.07-5.18)	0.97 (0.90-1.05)
	P-value^a^	Ref	0.794	0.633	0.485
	OR^b^ (95% CI)	Ref	0.96 (0.29-3.15)	0.27 (0.02-3.03)	0.97 (0.90-1.05)
	P-value^b^	Ref	0.949	0.290	0.445

**OR** = Odds ratio, **CI** = Confidence interval, Ref: Reference

a Unadjusted OR.

b Adjusted for age, sex, season of blood draw, body mass index, hypertension, diabetes, history of smoking and history of alcohol intake.

Furthermore, a protective association was detected between plasma 25 (OH) D and tumor stage [25 (OH) D insufficiency 0.18 (95% CI: 0.04-0.79; P = 0.024)]. In addition, no significant association was observed among the cases with metastasis compared with the cases without metastasis [25 (OH) D insufficiency 0.96 (95% CI: 0.29-3.15; P = 0.949); normal 25 (OH) D level 0.27 (95% CI: 0.02-3.03; P = 0.290)] (Table-3).

## DISCUSSION

Vitamin D is a fat-soluble secosteroid, which is beneficial for enhancing the intestinal absorption of numerous key nutrients such as calcium, magnesium, iron and phosphate (4). Accumulating evidence suggests that there is a high prevalence of low vitamin D levels worldwide; the explanation for this phenomenon may involve the insufficient supply of vitamin D from natural food sources, a shift to sedentary lifestyles and limited outdoor activity (12). Vitamin D plays vital roles in intestinal, skeletal and other biological pathways, such as those in immune cells and the tumor microenvironment (cell proliferation, differentiation and apoptosis) (13). Some laboratory studies have reported that vitamin D is an anticarcinogenic agent. Few studies have been conducted on the association of plasma vitamin D with RCC, though the kidney is a major organ in vitamin D metabolism and activity (14, 15). Furthermore, the relationships between plasma vitamin D and RCC risk may not be easily generalized across geographical regions (e.g. China, Africa and Latin America).

To the best of our knowledge, there have been few studies on the association of plasma 25 (OH) D levels with RCC risk in the Chinese population. In our study, the inclusion criterion was patients diagnosed with RCC and treated with surgery. These RCC cases were matched with the same number of controls of a similar age, gender and ethnicity with measured plasma 25 (OH) D levels and no history of cancer. In this case-control study of Chinese adults, the circulating levels of 25 (OH) D were significantly decreased in RCC cases compared with controls. Furthermore, an inverse association was observed between plasma 25 (OH) D levels and RCC, indicating that lower circulating levels of 25 (OH) D were associated with an increased risk of RCC. The overall result was consistent with our previous meta-analysis. The previous pooled analysis included two prospective cohort studies and seven nested case-control studies, involving a total of 130.609 participants and 1.815 cases of kidney cancer. The combined results indicated that a higher level of circulating 25 (OH) D could reduce the risk of kidney cancer by 21% (OR = 0.79, 95% CI: 0.69-0.91) (8). Furthermore, our findings reproduce the well-characterized relationship between plasma 25 (OH) D levels and RCC, increasing the power and confidence in the results.

Circulating vitamin D is hydroxylated in the liver to hydroxyvitamin D (25 (OH) D), followed by the conversion of 25 (OH) D into 1.25-dihydroxy vitamin D (1.25 (OH) 2D), the active form of vitamin D, by CYP27B. The kidney can hydroxylate 25 (OH) D to its active form, which binds to the vitamin D and retinoid X receptors (4, 16, 17). The underlying mechanisms of the association between circulating 25 (OH) D and RCC are unclear. Several plausible biological links might explain the association between plasma 25 (OH) D and the risk of RCC. The anti-cancer effects of 25 (OH) D have been demonstrated in in vitro and animal studies (18), and the protective roles of 25 (OH) D against cancer may involve decreasing the expression of aromatase and suppressing tumor angiogenesis, invasion and metastasis (19, 20). Recent studies have shown that vitamin D could reduce the incidence of colorectal, breast and prostate cancer through interactions with some important pathway proteins, such as insulin like growth factor-1 (IGF-1) and IGF-binding protein 3 (21–24). Vitamin D may also interact with some established risk factors for kidney cancer, such as BMI, smoking, hypertension and diabetes (25–27). Furthermore, the results of some other studies are different from ours. Gallicchio et al. do not support the hypothesis that higher circulating 25 (OH) D is associated with a decreased risk of kidney cancer overall, or with RCC specifically, on the basis of pooling eight prospective cohort studies (28). Therefore, vitamin D may still have some unknown effects against cancer (8).

A major strength of the present study is that it is the first case-control study on the relationship between circulating levels of 25 (OH) D and RCC risk in China. We included newly diagnosed RCC cases and assessed plasma 25 (OH) D status from blood samples collected prior to surgery. The circulating 25 (OH) D concentrations were examined in a single laboratory in our hospital to reduce interlaboratory variation. Because our specimens were measured randomly in the laboratory, it is highly unlikely that these measurements differed between cases and controls. Furthermore, our results were robust when the well-known risk factors for RCC, such as age, BMI and smoking, were adjusted in our analysis, which allows us to make a more accurate estimate of the association between plasma 25 (OH) D levels and RCC risk.

Our study has several limitations that need to be taken into account. First, the measurements relied on only a single blood sample, which may result in a lack of precision. Second, circulating levels of 25 (OH) D in participants may be altered by their lifestyle, such as physical activity and dietary patterns, which may be a source of bias in this study. Although some confounding factors were adjusted in our analysis, we could not rule out the possibility that other unidentified or unmeasured factors could affect the association. Third, the study design was case-control; such studies are inclined towards bias, and the sample size was limited. In addition, the study sample comprised only a Chinese Han population, which may limit the application of these results to more diverse populations. Therefore, large prospective cohort observational studies including diverse populations are needed in the future.

To sum up, the findings of our case-control study on a Chinese Han population showed that the circulating 25 (OH) D level has an independent inverse association with the risk of RCC, whether confounding factors are adjusted or not.
